# Authentication of Processed *Epimedii folium* by EA-IRMS

**DOI:** 10.1155/2020/8920380

**Published:** 2020-02-03

**Authors:** Fengyan He, Mengyi Li, Yi He, Zhe Dong, Jin Cao, Zhong Dai, Shuangcheng Ma

**Affiliations:** National Institutes for Food and Drug Control, Beijing 100050, China

## Abstract

Processing of crude drug is a key character of traditional Chinese medicine (TCM), which could enhance the efficacy and/or reduce the toxicity of crude drugs to fulfill different requirements of TCM clinical practice. *Epimedii folium* (EF) is a widely used TCM. As a traditional method of TCM, EF is processed with refined mutton fat before being used in clinical practice. It has been reported that processing EF with mutton fat could improve the bioavailability and intestinal absorption of epimedium flavonoids and thus enhances the pharmacological effects. For economic benefits, it is possible to adulterate processed EF with unprocessed drug or process EF with cheaper plant oils. In the present study, 17 batches of crude and processed EF samples were collected from the Chinese market and 10 batches of replica processed drugs were prepared with different edible plant oils and animal fats in our laboratory. Elemental analyzer coupled with isotopic ratio mass spectrometry (EA-IRMS) was applied to determine the *δ*^13^C values of the cyclohexane extracts of those samples. Significant differences could be observed in the results. EA-IRMS could be used to discriminate raw EF, processed EF, and EF processed with C3 plant oils.

## 1. Introduction

As a widely used traditional Chinese medicine (TCM), *Epimedii folium* (EF) is the dried leaves of *Epimedium brevicomu* Maxim, *E. sagittatum* (Sieb. et Zucc.), *E. pubescens* Maxim, or *E. koreanum* Nakai [1]. EF was first recorded in *Shen Nong Ben Cao Jing*, an ancient book of TCM written about 2000 years ago [2]. In China, EF has been used as a tonic, antirheumatic, and aphrodisiac for thousands of years. Phytochemical and pharmacological investigations revealed that flavonoids were the main bioactive constituents of this drug [3–6].

Processing of crude drug is an important character of TCM [7]. Based on clinical practice and TCM theory, the processing could enhance the efficacy and/or reduce the toxicity of crude drugs. The processing method of EF listed on Chinese Pharmacopoeia was stir-frying with refined mutton fat. For each 100 kg EF, 20 kg refined mutton fat was used during the processing [1]. This method was first recorded in an ancient classic of TCM processing named *Lei Gong Pao Zhi Lun* (about 500 AD). Refined mutton fat was the fried fat of *Capra hircus* Linnaeus or *Ovis aries* Linnaeus. Refined mutton fat is “warm” and possesses the effects of “restoring deficiency,” “moistening dryness,” “dispelling wind,” and “removing toxin,” according to TCM theories [2]. Although raw and processed EF both have “antirheumatic” and “bone-strengthening” effects, it is believed that the processed product has better effect on “tonifying kidney.” Modern pharmacological investigation has demonstrated that processing with refined mutton fat could improve the bioavailability and intestinal absorption of epimedium flavonoids and enhance the pharmacological effects of EF [8–11]. Therefore, stir-frying with refined mutton fat was essential for the clinical usage of EF.

Refined mutton fat was also a commonly used edible fat in Chinese diet. Its price was higher than normal plant oils such as rape oil and soybean oil. Economic benefit makes it possible to adulterate processed EF with raw materials or process EF with cheaper plant oils. In consideration of the improved pharmaceutical activities of processed EF, it is necessary to establish a reliable method to discriminate raw, processed drugs and drugs processed with other plant oils.

In Chinese Pharmacopeia, the olfactory sensation of refined mutton fat was used for the authentication of processed EF [1]. No other method has been reported so far. Branched chain fatty acids (BCFAs) were considered as main determinants of the odor of refined mutton fat. Several methods have been reported for the determination of these components by gas chromatography (GC) or gas chromatography coupled with mass spectrometry (GC-MS) [12, 13]. BCFAs were of relative volatility and in low concentration levels in many animal fats and could be influenced by age, gender, breed, and nutrition of animals [14]. Furthermore, the BCFAs might evaporate during mutton fat refining, drug processing, transport, and storage of processed EF. Thus, BCFAs were not ideal markers for the authentication of processed EF.

Isotopic ratio mass spectrometry (IRMS) is a special mass spectrometry technique which measures the relative abundance of isotopes of light elements [15]. Elemental analyzer coupled with IRMS (EA-IRMS) is a generally used IRMS interface which determines the average isotopic signal of the entire sample. Compared with other IRMS interfaces such as gas chromatography/combustion coupled with IRMS (GC/C/IRMS), EA-IRMS is fast and simple, without complicated sample pretreatment. EA-IRMS has been applied in the authenticity evaluation and geographical traceability of herbal drugs and foods especially in the characterization of plant oils and animal fats [16–19]. It has been reported that edible vegetable oil and animal fat possessed different *δ*^13^C values [18, 19]. Because the refined mutton fat is the main part of the nonpolar extract of processed EF, it is possible that the nonpolar extracts of the raw and processed EF have different *δ*^13^C values. In the present study, 17 batches of raw and processed EF were collected, 1 batch of EF raw material was processed with different vegetable oils and animal fats in our laboratory, and the *δ*^13^C values of those samples were analyzed by EA-IRMS to discriminate raw and processed EF and the oils used for the processing of EF.

## 2. Materials and Methods

### 2.1. Sample Collection

Seventeen batches of EF samples were collected from market, including 8 batches of raw materials (No. 1–8) and 9 batches (No. 9–17) of processed drugs. In those samples, Sample Nos. 2, 3, and 7 were identified to be the leaves of *Epimedium koreanum* Nakai, and the rest samples were the leaves of *E. brevicomu* Maxim by Prof. Nanping Zhang (National Institutes for Food and Drug Control, Beijing, China).

### 2.2. Preparation of Replica Processed Drugs

To evaluate the influence of adjuvants on the *δ*^13^C value of processed EF, raw material No. 6 was processed with different plant oils and animal fats in our laboratory to obtain samples R1–R10. Plant oils included C3 plant oils (soybean oil (Soy), sunflower oil (Sun), rapeseed oil (Rap), and peanut oil (Pea)) and C4 plant oil (maize oil (Mai)). Three batches of mutton fats were used and labeled as Mut A, Mut B, and Mut C. As common edible fats, pork fat (Por) and beef fat (Bee) were chosen to be the possible adulterations of mutton fat. Plant oils were purchased from a local supermarket. Animal fats were fried with animal adipose tissues in our laboratory. The processing followed the method of Chinese pharmacopeia.

### 2.3. Extraction with Cyclohexane

The samples (raw materials, processed drugs, and replica processed drugs) were pulverized to powder. Then, 1 g of the drug powder was ultrasonically extracted with cyclohexane (analytical grade, Sinopharm Chemical Reagent Co. Ltd, Beijing, China) under ice bath for 30 min. After filtration, the extract was dried under reduced pressure at 40°C. The residuals were stored in fridge at 4°C before use.

### 2.4. *δ*^13^C Determination

The *δ*^13^C values of extracts and bulk adjuvant were measured using Thermo Flash 2000-HT elemental analyzer (Thermo Fisher Scientific, Bremen, Germany) coupled with a Thermo Delta-V Advantage isotope ratio mass spectrometer (Thermo Fisher Scientific) via a Thermo DELTA/MAT253 ConFlo IV Interface (Thermo Fisher Scientific). The elemental analyzer was controlled by Finnigan Eager 300 Isodat software (version 3.0). The oxidation and reduction reactors were heated to 960°C, respectively. The oven temperature was set at 50°C. The carrier gas (He) flow was about 90 mL/min. The flow rate of O_2_ gas flash combustion was 180 mL/min. The run time of the experiment was approximately 5 min for a single run.

The carbon isotopic composition was expressed as the difference (*δ*) between an analyte and an international standard, Vienna Pee Dee Belemnite (VPDB), based on the following equation:

(1) $$\begin{array}{c} \delta^{13}\text{C}=\left(\frac{R_{\text{sample}}}{R_{\text{standard}}}-1\right)\times 1000, \end{array}$$

where *R*_sample_ is the isotope ratio measured for the sample and *R*_standard_ is the isotope ratio of international standard. The delta values are expressed in units “per mil” (‰). Analysis of each sample was performed in triplicate. The isotopic values were calculated against international reference material urea (IVA33802174, *δ*^13^C = −43.26‰, IVA analysentechnik E. K., Germany). For calibration, the reference material was analyzed every 10 samples. As precision of analysis, the standard deviation (SD) of calculated urea results was below 0.15‰ and 0.2‰ for sample repeats.

### 2.5. Statistical Analysis

One-way ANOVA was performed on the cyclohexane extract amounts of drug samples. Independent samples *T* test was conducted on *δ*^13^C values of cyclohexane extracts from raw and processed drugs. Pearson correlation analysis was performed on *δ*^13^C values of cyclohexane extracts of replica processed drugs and corresponding adjuvants. SPSS software (version 16.0) was used.

## 3. Results and Discussion

### 3.1. Yields of Cyclohexane Extracts

The yields of cyclohexane extracts of 8 batches of EF raw materials and 9 batches of purchased processed products ranged from 0.31 to 1.11% and 9.04 to 32.26%, respectively ([Supplementary-material supplementary-material-1]). For replica processed drugs, the yields ranged from 10.22 to 15.21% ([Supplementary-material supplementary-material-1]). It suggested that the nonpolar component amounts of processed products, either purchased from the Chinese market or reproduced in our laboratory, were significantly higher than that of the raw materials (one-way ANOVA, *p* < 0.05). The difference between cyclohexane extracts amounts of processed drugs and the replica processed drugs were not significant (one-way ANOVA, *p* > 0.05). Therefore, the nonpolar component amount was influenced greatly by the processing procedure and could be used as a marker for discrimination of raw and processed EF.

Compared with replica processed drugs, the nonpolar component amounts of purchased processed drugs ranged greatly. This might be attributed to the variety of specific processing parameters in different processing factories and probably heterogeneous distribution of refined mutton fat in bulk drugs.

### 3.2. *δ*^13^C Determination of Cyclohexane Extracts of Collected Samples

**Figure 1:**
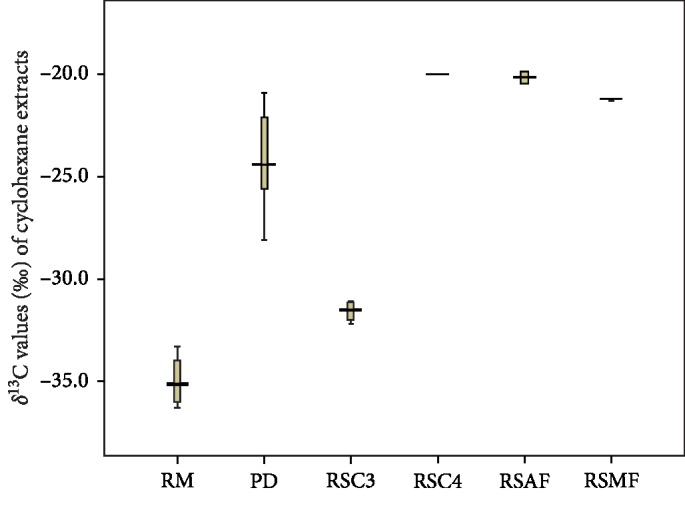
Boxplot of *δ*^13^C values of cyclohexane extracts from raw materials (RM), processed drugs (PD), replica samples processed with C3 plant oils (RSC3), replica samples processed with C4 plant oils (RSC4), replica samples processed with other animal fats (RSAF), and replica samples processed with mutton fats (RSMF).

The *δ*^13^C values of 8 batches of raw materials and 9 batches of processed drugs ranged from −36.3 to −33.3 ‰ and from −28.1 to −20.9‰ ([Supplementary-material supplementary-material-1]). As shown in Figure 1, the *δ*^13^C values of processed drugs are significantly higher than those of raw materials (independent samples *T*-test, *p* < 0.05). *δ*^13^C value of cyclohexane extract could be used as a key marker to distinguish EF raw materials and processed drugs.

### 3.3. *δ*^13^C Determination of Adjuvants and Cyclohexane Extracts of Replica Processed Samples

Plant oils of Pea, Soy, Sun, and Rap possess *δ*^13^C values ranging from −31.4 to −30.9‰, while Mai shows the *δ*^13^C value of −17.5‰ ([Supplementary-material supplementary-material-1]). The results were consistent with other reports about the *δ*^13^C values of C3 and C4 plants oils [19, 20].

For animal fats, Por had the highest *δ*^13^C value of −17.4‰, while Bee and Mut A, B, and C had the *δ*^13^C values nearly equal to −19.0‰ (−19.0, −19.2, −18.9, and −19.6‰, [Supplementary-material supplementary-material-1]).

The *δ*^13^C value of replica processed drugs made with Pea, Soy, Sun, and Rap oils ranged from −32.2 to −31.1‰, and the *δ*^13^C value of those processed with Mai and 5 animal fats ranged from −21.3 to −19.9‰ ([Supplementary-material supplementary-material-1]).

Pearson correlation analysis revealed that *δ*^13^C values of adjuvants were significantly correlated to *δ*^13^C values of cyclohexane extracts of replica processed drugs (coefficient = 0.998), which revealed that oil or fat used during processing dominated the *δ*^13^C value of cyclohexane extract for processed drugs.

It is well known that plants can be divided into C3 and C4 plants based on the difference of photosynthetic pathways. Most of the plants were C3 plants, expecting about 8100 species including the food crops maize, sugar cane, millet, and sorghum [21, 22]. C4 plants have higher natural ^13^C enrichment than C3 plants. This is the reason why Mai shows the more positive *δ*^13^C value than other C3 plant oils.

The *δ*^13^C values of plant oils were mainly dominated by photosynthetic pathways and were relatively constant. For the adulterant processed with C3 plant oils, the *δ*^13^C value could be used as a prominent marker. But for adulterant processed with C4 plant oils (such as maize oil), blend oils including C3 and C4 oils, or other common edible animal fats, the current method was not adequate. It should be further studied.

### 3.4. *δ*^13^C Values of EF Processed with Animal Fats

In China, Sheep and goats were raised in a large scale including west plateaus, east, and middle plains [23]. It has been revealed that *δ*^13^C values of animal tissues were close related to the feed regime [24–26]. Feeding regimes of sheep and goat were different from place to place. More pastures would be feed in the pastoral area, and more crop by-products would be used in the crop area. Besides, silage, straw, concentrate feed, bran, soybean cake, cottonseed cake, and other feed were also generally used. Difference between feed regimes for sheep and goats should be the dominant factor for the varieties of the *δ*^13^C value of cyclohexane extracts of purchased processed drugs. Only the livestock feed with natural pastures (mostly C3 plants) could possess *δ*^13^C values close to EF raw materials [25]. However, in view of the high farming cost, the fats of pasture-fed livestock were less likely to be used as processing adjuvant. This assumption was also confirmed by the detected *δ*^13^C values of processed EFs ([Supplementary-material supplementary-material-1]).

It should been noticed that maize (maize, silage maize, maize straw, or concentrated feed made with maize) was widely used in livestock production in China. As shown in [Supplementary-material supplementary-material-1], all drugs processed with animal fats show the *δ*^13^C values between C3 and C4 plant oils. A maize-based diet for different livestock also leaded to similar *δ*^13^C values of different animal fats. Thus EA-IRMS is not adequate for the discrimination of drugs processed with other animal fats.

On the other hand, the prices of pork, beef, and other animal fats were close to refined mutton fat, making the adulteration with animal fats less profitable than plant oils. Therefore, discrimination of the EF processed with other animal fats is not so urgent.

### 3.5. Optional Methods for the Authentication of Processed EF

According to our experiment results, together with reported traditional morphological characters, 3 methods could be suggested for the authentication of processed EF.

The first method is the morphological identification. According to Chinese Pharmacopeia, the processed EF should have a pale yellow surface with fatty luster and smells a slight odor of refined mutton fat, in contrast to the raw drug which is yellowish-green or greyish-green and with slight odor [1]. The changes in the morphological characters were the results of the used fat and the high temperature during processing. However, due to the diversities of mutton fat quality and processing parameters, the morphological identification should be based on personal professional experiences.

The amount of cyclohexane extract could also be used for the authentication of processed EF. As shown in [Supplementary-material supplementary-material-1], the cyclohexane extract amount ranged from 9.04% to 32.26% for processed drugs and 0.31 to 1.11% for raw materials. The difference between the cyclohexane extract amounts of raw and processed EFs was significant (*p* < 0.01) and could be used for the identification of raw and processed EF. Because of the added fats, the cyclohexane extract amount was increased after processing. Nevertheless, if plant oils were used during the processing, the cyclohexane extract amount could also be increased ([Supplementary-material supplementary-material-1]).

The third authentication method is the *δ*^13^C value of cyclohexane extract. For processed EF, most of the cyclohexane extracts were the nonpolar components from the oil or fat used during processing. Thus, its *δ*^13^C value was corresponding to the used oil or fat, which was consistent with our study ([Supplementary-material supplementary-material-1]). The different *δ*^13^C values of C3 plant oils and animal fats could be used to estimate the excipients used for EF processing.

## 4. Conclusion

In the present study, EA-IRMS was used for discrimination of raw, processed EF, and the oil used during processing. Cyclohexane extract amount and its *δ*^13^C values were significantly different between the raw and processed EF. The drugs processed with C4 plant oils or other animal fats show the similar *δ*^13^C values with the drug processed with refined mutton fat. However, the drugs processed with C3 plant oils possess more negative *δ*^13^C values than the drugs processed with refined mutton fat, as described in Chinese Pharmacopeia. EA-IRMS could be applied to discriminate raw EF, processed EF, and the C3 plant oils used during EF processing. The EA-IRMS methods have been established for the authenticity evaluation and geographical traceability of foods, including plant oils and edible animal fats [18–20]. However, few papers have reported about the application of EA-IRMS on the authentication of processed Chinese material medica. The application of EA-IRMS on the quality control of traditional Chinese medicine possesses unique advantages and should be paid more attention.

## Data Availability

The data used to support the findings of this study are included within the article.

## Supplementary Materials

Supplementary Materials Table S1: amounts and *δ*^13^C values (‰) of cyclohexane extracts from 17 batches of collected samples (*n* = 3). Table S2: amounts and *δ*^13^C values (‰) of cyclohexane extracts from 10 replica processed drugs and *δ*^13^C values (‰) of corresponding adjuvant (*n* = 3).
