# Supplementary material for: Authentication of Processed Epimedii folium by EA-IRMS
Source: J Anal Methods Chem. 2020 Feb 3;2020:8920380. doi: 10.1155/2020/8920380 (PMC7023830; doi:10.1155/2020/8920380)
Supplement: Supplementary Materials — Table S1: amounts and δ13C values (‰) of cyclohexane extracts from 17 batches of collected samples (n = 3). Table S2: amounts and δ13C values (‰) of cyclohexane extracts from 10 replica processed drugs and δ13C values (‰) of corresponding adjuvant (n = 3). [file 8920380.f1.docx]

**Appendix A.** Supplementary data

**Authentication of processed *Epimedii folium* by EA-IRMS**

**Fengyan He ∣Mengyi Li^†^ ∣Yi He ∣Zhe Dong ∣Jin Cao∣Zhong Dai∣Shuangcheng Ma**

National Institutes for Food and Drug Control, Beijing 100050, China

^†^- This author has contributed equally to this work.

**Correspondence**

Yi He and Shuangcheng Ma, National Institutes for Food and Drug Control, Beijing 100050, China.

Email: zjsheyi@sina.com; shuangcma@hotmail.com

**TABLE S1** Amounts and δ^13^C values (‰) of cyclohexane extracts from 17 batches of collected samples (n=3).

| Sample type | Sample number | Extract amount (%) | Average amount (%) | δ^13^C values (‰) | Average δ^13^C values (‰) |
| --- | --- | --- | --- | --- | --- |
| raw material | 1 | 0.75 | 0.87±0.28 | -34.1 | -35.0±1.1 |
|  | 2 | 0.63 |  | -33.9 |  |
|  | 3 | 0.31 |  | -33.3 |  |
|  | 4 | 1.00 |  | -35.5 |  |
|  | 5 | 0.99 |  | -36.3 |  |
|  | 6 | 1.08 |  | -36.0 |  |
|  | 7 | 1.05 |  | -36.0 |  |
|  | 8 | 1.11 |  | -34.8 |  |
| processed drug | 9 | 10.59 | 18.74±8.24 | -25.6 | -24.1±2.2 |
|  | 10 | 32.26 |  | -28.1 |  |
|  | 11 | 16.56 |  | -21.9 |  |
|  | 12 | 28.11 |  | -24.4 |  |
|  | 13 | 21.96 |  | -20.9 |  |
|  | 14 | 11.31 |  | -22.1 |  |
|  | 15 | 14.92 |  | -23.9 |  |
|  | 16 | 9.04 |  | -25.7 |  |
|  | 17 | 23.88 |  | -24.7 |  |

**Table S2.** Amounts and δ^13^C values (‰) of cyclohexane extracts from 10 replica processed drugs and δ^13^C values (‰) of corresponding adjuvant (n=3).

| sample number | Adjuvant used | δ^13^C values (‰)  of adjuvant | Extract amount (%) | δ^13^C values (‰)  of processed EF |
| --- | --- | --- | --- | --- |
| R1 | Pea | -30.9 | 11.11 | -31.1 |
| R2 | Soy | -31.4 | 12.21 | -32.2 |
| R3 | Sun | -31.2 | 11.83 | -31.8 |
| R4 | Rap | -30.9 | 10.53 | -31.2 |
| R5 | Mai | -17.5 | 11.49 | -20.0 |
| R6 | Por | -17.4 | 10.22 | -19.9 |
| R7 | Bee | -19.0 | 14.25 | -20.4 |
| R8 | Mut A | -19.2 | 12.64 | -21.2 |
| R9 | Mut B | -18.8 | 13.48 | -21.2 |
| R10 | Mut C | -19.6 | 15.21 | -21.3 |
